# Association Between the Built Environment in School Neighborhoods With Physical Activity Among New York City Children, 2012

**DOI:** 10.5888/pcd13.150581

**Published:** 2016-08-18

**Authors:** Matthew M. Graziose, Heewon Lee Gray, James Quinn, Andrew G. Rundle, Isobel R. Contento, Pamela A. Koch

**Affiliations:** Author Affiliations: Heewon Lee Gray, Isobel R. Contento, Pamela A. Koch, Teachers College Columbia University, New York, New York; James Quinn, The Institute for Social and Economic Research and Policy Built Environment and Health Working Group, Columbia University, New York, New York; Andrew G. Rundle, Columbia University Mailman School of Public Health, New York, New York.

## Abstract

**Introduction:**

The benefits of physical activity for health and well-being are well established, yet built environment characteristics in the school neighborhood may constrain students’ ability to engage in physical activity and contribute to the considerable variation in physical activity among students at different schools.

**Methods:**

Baseline data from the Food, Health and Choices obesity prevention trial were used to create multilevel linear models of the relationship between fifth-grade students’ (n = 952) physical activity and related psychosocial factors and characteristics of the built environment of the school’s neighborhood (park access, public transportation density, total crime, and walkability), controlling for age and body mass index *z* scores.

**Results:**

Total crime was inversely associated with boys’ light physical activity duration (β = −0.189; *P* = .02) and behavioral intention for physical activity (β = −0.178; *P* = .03). Boys’ habit strength for physical activity was positively associated with public transportation density (β = 0.375; *P* = .02) and negatively associated with total crime (β = −0.216; *P* = .01), explaining 67% of between-school variation. Girls’ frequency of light physical activity was positively associated with park access (β = 0.188; *P* = .04). Built environment characteristics explained 97% of the between-school variation in girls’ self-efficacy in walking for exercise.

**Conclusions:**

Characteristics of the built environment surrounding schools were associated with and explain between-school variation in students’ physical activity and several theory-based psychosocial factors. Partnerships between public health practitioners, policy makers, and school administrators may be warranted to shape the school neighborhood, specifically to decrease crime rates and increase park access, to encourage physical activity in youth.

## Introduction

Many children fail to meet physical activity (PA) recommendations set forth by federal agencies, increasing their risk for overweight, obesity, and chronic disease (1,2). In New York City (NYC), the location of our study, the prevalence of obesity mirrors the national average, with 1 in 5 children considered obese (3). Schools are often the setting of obesity prevention programs that seek to increase PA, yet these programs may fail to account for barriers students face to engaging in PA outside of school or as they travel to and from school (4).

Theoretical and empirical evidence links aspects of the built environment to PA (4,5). Characteristics of a neighborhood such as residential density, walkability, land-use patterns, and park access are associated with PA among children (6,7). Many studies, however, operationalize the neighborhood as the area surrounding the home and thus may not be useful in informing policy to support PA around schools. Moreover, children’s PA behaviors often vary considerably from school to school (8,9), especially in NYC (10), potentially resulting from characteristics of the neighborhood’s built environment.

An understanding of how built environment characteristics relate to PA and explain between-school variability can contribute to the design and evaluation of childhood obesity prevention programs. Interventions that target a child’s individual, school, and environmental determinants of PA jointly are likely more effective than those focusing on just 1 determinant (4,11). Interventions that address theory-based psychosocial mediators of behavior are also more likely to change behavior (11), yet few studies have examined built environment effects on psychosocial mediators of PA in children. We focus on 3 psychosocial factors (self-efficacy, behavioral intentions, and habit strength) that have empirical support as mediating variables in intervention studies (11). Our assumption was that these factors mediate the relationship between the environment (or an intervention) and students’ PA behavior.

We examined the association between built environment characteristics in the school neighborhood (park access, public transportation density, total crime, and walkability) and self-reported PA behavior and related theory-based psychosocial factors in a sample of urban fifth-grade public school students in NYC. We also assessed the proportion of between-school variation in PA and related psychosocial factors explained by these characteristics.

## Methods

This cross-sectional study uses baseline data from Food, Health and Choices (FHC), a cluster-randomized controlled trial to prevent obesity, collected between September 2012 and January 2013. All fifth-grade students (n = 1,387) from 20 public elementary schools, mainly in low-resource neighborhoods, were invited to participate. Students were enrolled through passive consent, and parents could opt to remove them from the study. Of enrolled students, 1,241 (89.5%) completed a questionnaire measuring PA behaviors and psychosocial factors and 1,165 (84.0%) completed anthropometric measurements. Students who were deemed by Tukey’s procedure (12) to be outliers on the basis of body fat data (n = 5) and students older than 14 years (n = 1) were excluded from the analyses. Other students had missing data because of absenteeism during data collection. Students with both anthropometric and questionnaire data (n = 952) were included in this analysis. The Teachers College Columbia University and New York City Department of Education (NYCDOE) institutional review boards approved this study.

### Students’ PA behaviors and psychosocial factors

Students completed the FHC questionnaire (FHC-Q) (13), using an audience response system, to assess frequency and duration of PA. The questionnaire included 3 PA frequency questions, 1 per light, medium, and heavy intensity (light intensity, “In the past week, I did things that got me up and moving”; medium intensity, “In the past week, I did things that made my heart beat a little faster”; and heavy intensity, “In the past week, I did things that got my heart beating really fast”). Response options were 1, 0 times per week; 2, about 1 to 2 times per week; 3, about 3 to 4 times per week; 4, almost every day; and 5, 2 or more times every day. There were 3 duration questions, 1 per light, medium, and heavy duration (light duration, “How long each time did I do things that got me up and moving?”; medium duration, “How long each time did I do things that made my heart beat a little faster?”; and heavy duration, “How long each time did I do things that got my heart beating really fast?”). Response options were 1, less than 0.5 hour; 2, 0.5 to 1 hour; 3, 2 hours; 4, 3 hours; 5, more than 3 hours. We summed the 3 respective items to create scales to reflect overall frequency and duration of PA. Internal consistency, assessed by Cronbach’s α, was 0.63 for PA frequency and 0.78 for PA duration.

The questionnaire also assessed perceptions of self-efficacy (“I am sure I can walk or bike to school instead of taking a car, bus, or subway” and “I am sure I can walk for exercise”), behavioral intention (“I would like to do more physical activity”), and habit strength (“When I think about myself, physical activity is part of my daily routine”). These items are based on social cognitive and self-determination theories (14). Response options were on a Likert scale (1, not at all sure; 2, a little sure; 3, neither sure or not sure; 4, sure; 5, very sure; or 1, not at all true for me; 2, not true for me; 3, neither true or not true; 4, somewhat true for me; 5, very true for me).

### Students’ anthropometrics and school-level demographics

Students’ height (in centimeters) was measured with a portable stadiometer (Seca model 213) and weight (in kilograms) with a Tanita body composition analyzer (model SC-331s). Trained, graduate-level nutrition students collected measurements by using a standardized protocol in the morning before lunch, when possible, and collected all data for 1 school on the same day. Measurements were repeated twice or until 2 measures for height fell within 1.0 cm of each other and for weight within 0.1 kg of each other, and averaged. We calculated body mass index (BMI) (weight in kilograms divided by height in meters squared) and then calculated BMI-for-age percentile and BMI *z* score based on the Centers for Disease Control and Prevention growth charts (14) using birthdates obtained from school enrollment records.

To describe the student body we collected demographic data for each school through publicly available records from the NYCDOE for the 2012–2013 school year. These included percentage of students who were black, Hispanic, English language learners, and eligible for free or reduced-price lunch.

### Neighborhood built environment characteristics

Each elementary school in NYC is assigned an administrative area in which all residing school-aged children are guaranteed enrollment to the respective school. We used this area, called the “enrollment zone,” to define the neighborhood surrounding the schools in this study, producing 20 unique neighborhoods. We calculated 4 built environment characteristics (park access, public transportation density, total crime, and walkability) for the 20 school neighborhoods in our sample and for the population of NYC elementary school enrollment zones (n = 728) for comparison. We used ArcGIS version 10.2 (ESRI) for geospatial analysis.

We obtained park and playground data from the NYC Department of Parks and Recreation current as of 2012. Park access was calculated as the total land area as parks or playgrounds divided by the total land area of each enrollment zone in square kilometers. Public transportation density was calculated as a count of all unique bus and subway stops (obtained from the NYC Metropolitan Transport Authority and current as of 2012) divided by the total land area of each enrollment zone. Total crime was calculated as an area-weighted average for each enrollment zone using CrimeRisk Index variables from 2012 acquired from Esri (www.esri.com/data/esri_data/business-overview/crimerisk) at the 2010 census block group level. Total crime index was personal crime, murder, rape, robbery, assault, property crime, burglary, larceny, and motor vehicle theft; a value of 100 on this index represents the national average. The walkability index, adapted from Frank et al (15), was calculated as the sum of the *z* scores of 4 environmental measures: land use mix (an entropy measure of land use based on the distribution of building floor area among 6 land use types: education, entertainment, single-family residential, multifamily residential, retail, and office), intersection density (unique intersections per square kilometer), residential population density (residential units per square kilometer) and retail floor area density (retail floor area per square kilometer). We obtained land use mix and retail floor area density from the Primary Land Use Tax Lot Output from the NYC Department of Planning current as of 2011. Residential population density was calculated for each neighborhood by spatially intersecting the enrollment zone with 2010 US Census tracts and interpolating residential population counts from the 2008–2012 American Community Survey within each tract matched to the enrollment zone geography using an aerial-weighting method to transfer data from one set of spatial units to another. We then divided the total apportioned residential population by the total land area within each enrollment zone. Intersection density was calculated using a street centerline geographic information system layer from the New York State Accident Location Information System. All 4 of these measures of built environment characteristics were converted to *z* scores and summed to calculate the walkability index. A higher score on the index represents a neighborhood more likely to support walking.

### Data analysis

We created multilevel linear models to account for cluster effects at the school level. First, we assessed the degree of clustering, or the proportion of variance attributable to the school level, by calculating intraclass correlation coefficients (ICCs) for each PA outcome. Next, using methods employed in previous school-based research (8,9) and recommendations from other researchers (16), we constructed models in a series of steps to assess model fit, separately for boys and girls, controlling for age and BMI *z* score as potential confounders.

Model 1 examined the between-school variation in individual students’ PA behaviors and psychosocial factors without any explanatory variables and was used to calculate baseline ICCs. Model 2 examined the association between school-level demographic variables (free or reduced-price lunch eligibility, black or Hispanic race/ethnicity, and English language learners) and PA behaviors and psychosocial factors. Model 3 was a full model that used all 4 built environment characteristics and controlled for age and BMI *z* score. Model 4 was a reduced model, constructed by using stepwise exclusion of nonsignificant school-level effects from the previous model.

We transformed each variable by dividing by the interquartile range (IQR) (75th percentile minus 25th percentile) to ensure comparability across built environment characteristics (3). β coefficients for each built environment characteristic can be interpreted as the association between the difference in the IQR of that variable. For each model, we report the β coefficient and *P* value for each variable. To facilitate comparison between models, we report the deviance statistic (minus 2 times the natural logarithm of the likelihood) as an indicator of model fit (a smaller deviance is desirable). We also report the proportion of total between-school variance explained for each model, interpretable as the fraction of explainable variation at the school level that is explained by the model. All models were created using HLM version 7.0 (Scientific Software International, Inc), and missing data were excluded casewise when running the analysis.

## Results

Of the students in our study, 51% were female, and the students were an average of 10.6 years old (Table 1). In the schools, 59% of the students were Hispanic, 30% were black, and 86% were eligible for free or reduced-price lunch (Table 2). In the neighborhoods surrounding these schools, park access, public transportation density, and walkability were all slightly higher than the average for all NYC schools.

**Table 1:** Characteristics of Participants in a Study of Associations of the Built Environment in School Neighborhoods With Physical Activity, New York City, 2012

Characteristic	Boys (n = 468)		Girls (n = 484)	
	Mean (SD)	ICC	Mean (SD)	ICC
**Age, mo**	127.06 (6.75)	—	126.20 (6.40)	—
**Body mass index (*z* score)**	0.77 (1.20)	—	0.58 (1.20)	—
**Physical activity frequency^a^**	10.33 (3.28)	0.049	9.67 (3.49)	0.065
Light	3.58 (1.35)	0.023	3.67 (1.27)	0.081
Medium	3.57 (1.37)	0.080	3.35 (1.37)	0.045
Heavy	3.86 (1.32)	<0.001	3.33 (1.39)	0.008
**Physical activity duration^b^**	9.52 (3.83)	0.048	8.30 (3.68)	0.061
Light	3.22 (1.51)	0.020	2.91 (1.43)	0.058
Medium	3.35 (1.50)	0.069	2.87 (1.39)	0.067
Heavy	3.73 (1.47)	0.024	3.04 (1.46)	0.010
**Self-efficacy of walking for exercise^c^**	4.37 (1.13)	0.003	4.44 (1.02)	0.011
**Self-efficacy of walking or biking instead of taking a car, bus, or subway to school^c^**	4.16 (1.42)	0.011	4.04 (1.45)	0.044
**Behavioral intention^d^**	4.33 (1.16)	0.068	4.33 (1.13)	0.055
**Habit strength^d^**	3.82 (1.46)	0.027	3.94 (1.35)	0.029

Abbreviations: —, not applicable; ICC, intraclass correlation coefficient; SD, standard deviation.

a Assessed with the questions: light frequency, “In the past week, I did things that got me up and moving”; medium frequency, “In the past week, I did things that made my heart beat a little faster”; and heavy frequency, “In the past week, I did things that got my heart beating really fast.” Response options: 1, 0 times/wk; 2, about 1–2 times/wk; 3, about 3–4 times/wk; 4, almost every day; 5, ≥2 times every day.

b Assessed with the questions: light duration, “How long each time did I do things that got me up and moving?”; medium duration, “How long each time did I do things that made my heart beat a little faster?”; and heavy duration, “How long each time did I do things that got my heart beating really fast?” Response options: 1, < .05 h; 2, 0.5 h to 1 h; 3, 2 h; 4, 3 h; 5, >3 h.

c Assessed with the question “I am sure I can walk for exercise” or “I am sure I can walk or bike to school instead of taking a car, bus or subway.” Response options: 1, not at all sure; 2, a little sure; 3, neither sure or not sure; 4, sure; 5, very sure.

d Assessed with the question “I would like to do more physical activity” or “When I think about myself, physical activity is part of my daily routine.” Response options: 1, not at all true for me; 2, not true for me; 3, neither true or not true; 4, somewhat true for me; 5, very true for me.

**Table 2:** Characteristics of Schools in a Study of Associations of the Built Environment in School Neighborhoods With Physical Activity, Compared With All New York City Schools, New York City, 2012

Characteristic	Sample (n = 20) , Mean (SD)	All New York City Schools (n = 728),^a^ Mean (SD)
**School**		
Total land area, km^2^	0.6 (0.68)	1.0 (1.45)
Eligible for free or reduced-price lunch, %	86.0 (19.28)	82.3 (22.63)
Black, %	29.6 (22.00)	32.0 (29.97)
Hispanic, %	59.0 (23.16)	39.8 (26.32)
English language learners, %	16.6 (10.62)	13.4 (11.43)
**Neighborhood**		
Total crime^b^	112.2 (59.73)	118.9 (42.71)
Public transportation density^c^	40.3 (16.78)	29.3 (25.12)
Walkability^d^	1.4 (1.27)	0.5 (2.63)
Park access^e^	0.14 (0.13)	0.08 (0.11)

Abbreviation: SD, standard deviation.

a The means of neighborhood built environment characteristics represents the population of all New York City elementary schools (n = 803 based on 2012–13 school year for schools and n = 728 for neighborhoods, because of schools co-located in buildings).

b Total crime index was personal crime, murder, rape, robbery, assault, property crime, burglary, larceny, and motor vehicle theft, where 100 represents the national average.

c Counts of all unique bus and subway stops divided by the total enrollment zone area in square kilometers.

d Sum of *z* scores of land use mix, intersection density, residential population density, and retail floor area density, ranging from −14.19 to 13.20.

e Proportion of school neighborhood (enrollment zone) land area covered by parks and playgrounds, ranging from 0 to 1.

For girls, the lowest ICC was observed for heavy PA frequency (0.008) and the highest for light PA frequency (0.081) (Table 1), indicating that about 0.8% and 8.1%, respectively, of the total variation in these behaviors were found between schools. For boys, the lowest ICC was observed for heavy PA frequency (<0.001) and the highest for medium PA frequency (0.080).

Model 2, which examined school-level demographic variables (eligibility for free or reduced-price lunch, percentage of black or Hispanic students, and percentage of English language learners) and students’ PA behaviors and psychosocial factors, yielded no significant outcomes and is not reported. Models 3 and 4 produced significant associations between built environment characteristics and student PA behaviors and psychosocial factors (Table 3 [boys] and Table 4 [girls]).

**Table 3:** Associations Between Characteristics of the Built Environment in School Neighborhoods and Physical Activity Outcomes Among Boys (n = 468), New York City, 2012

Variable	Model No.^a^	Total Crime^b^	Public Transportation Density^c^	Walkability^d^	Park Access^e^	Between- School Variation Explained,^f^ %	Deviance^g^
		β (Standard Error)^h^ [*P* Value]					
**Physical Activity Outcomes**							
**Physical activity frequency** i	3	0.266 (0.228) [.28]	−0.633 (0.423) [.16]	−0.482 (0.609) [.44]	0.056 (0.228) [.81]	NS	2148
Light	3	−0.090 (0.093) [.29]	−0.104 (0.163) [.32]	−0.140 (0.228) [.52]	0.007 (0.087) [.92]	NS	1315
Medium	3	0.232 (0.094) [.03]	−0.386 (0.168) [.04]	−0.360 (0.240) [.16]	0.001 (0.090) [.99]	41	1350
	4	0.193 (0.088) [.04]	−0.428 (0.162) [.02]	—	—	47	1348
Heavy	3	0.099 (0.070) [.18]	−0.177 (0.119) [.16]	−0.160 (0.159) [.33]	0.020 (0.063) [.76]	NS	1350
**Physical activity duration^j^**	3	−0.143 (0.266) [.60]	−0.072 (0.475) [.88]	−0.673 (0.680) [.34]	0.024 (0.251) [.92]	NS	2192
Light	3	−0.216 (0.083) [.15]	−0.052 (0.145) [.73]	−0.305 (0.191) [.13]	0.055 (0.074) [.47]	82	1341
	4	−0.189 (0.076) [.02]	—	—	—	59	1336
Medium	3	−0.047 (0.122) [.71]	−0.055 (0.216) [.80]	−0.369 (0.312) [.26]	−0.032 (0.115) [.79]	NS	2192
Heavy	3	0.056 (0.092) [.55]	0.027 (0.160) [.87]	−0.230 (0.221) [.31]	0.004 (0.083) [.96]	14	1360
**Psychosocial Factors**							
**Self-efficacy of walking for exercise^k^**	3	−0.063 (0.064) [.44]	0.017 (0.108) [.98]	0.072 (0.163) [.77]	−0.001 (0.059) [.78]	NS	1159
**Self-efficacy of walking or biking instead of taking a car, bus, or subway to school^k^**	3	−0.004 (0.089) [.97]	−0.093 (0.144) [.53]	0.073 (0.214) [.74]	−0.054 (0.081) [.52]	NS	1360
**Behavioral intention for physical activity^l^**	3	−0.180 (0.085) [.05]	0.046 (0.015) [.77]	0.096 (0.225) [.68]	−0.112 (0.082) [.16]	13	1172
	4	−0.178 (0.074) [.03]		0.179 (0.736) [.40]		31	1167
**Habit strength for physical activity^l^**	3	−0.178 (0.076) [.03]	0.378 (0.013) [.01]	−0.246 (0.173) [.18]	0.005 (0.068) [.94]	99	1432
	4	−0.216 (0.078) [.01]	0.375 (0.138) [.02]	—	—	67	1428

Abbreviations: —, variable was not included in the model; NS, nonsignificant.

a Model 3 is the full model, which uses all 4 built environment characteristics of interest and controls for age and body mass index *z* score. Model 4 is the reduced model, which was constructed by using stepwise exclusion of nonsignificant effects from model 3.

b Total crime index was personal crime, murder, rape, robbery, assault, property crime, burglary, larceny, and motor vehicle theft, where 100 represents the national average.

c Counts of all unique bus and subway stops divided by the total enrollment zone area in square kilometers.

d Sum of *z* scores of land use mix, intersection density, residential population density, and retail floor area density, ranging from −14.19 to 13.20. A higher score on the index represents a neighborhood more likely to support walking.

e Proportion of school neighborhood (enrollment zone) land area covered by parks and playgrounds, ranging from 0 to 1.

f Percentage of variation in mean physical activity behaviors and psychosocial factors explained by neighborhood-level built environment characteristics, calculated as (between-school variation from model 1 minus between-school variation from model 3 OR model 4) divided by between-school variation from model 1. Model 1 examined the between-school variation in individual students’ physical activity behaviors and psychosocial factors without any explanatory variables.

g Deviance is defined as minus twice the natural logarithm of the likelihood and is used an indicator of model fit.

h β coefficient (standard error) estimated for a difference equivalent to a 1 interquartile range (75th percentile minus 25th percentile) change.

i Assessed with the questions: light intensity, “In the past week, I did things that got me up and moving”; medium intensity, “In the past week, I did things that made my heart beat a little faster”; and heavy intensity, “In the past week, I did things that got my heart beating really fast.”

j Assessed with the questions: light duration, “How long each time did I do things that got me up and moving?”; medium duration, “How long each time did I do things that made my heart beat a little faster?”; and heavy duration, “How long each time did I do things that got my heart beating really fast?”

k Assessed with the question “I am sure I can walk for exercise” and “I am sure I can walk or bike to school instead of taking a car, bus or subway.”

l Assessed with the question “I would like to do more physical activity” and “When I think about myself, physical activity is part of my daily routine.”

**Table 4:** Associations Between Characteristics of the Built Environment in School Neighborhoods and Physical Activity Outcomes Among Girls (n = 484), New York City, 2012

Variable	Model No.^a^	Total Crime^b^	Public Transportation Density^c^	Walkability^d^	Park Access^e^	Between- School Variation Explained,^f^ %	Deviance^g^
		β (Standard Error)^h^ [*P* Value]					
**Physical Activity Outcomes**							
**Physical activity frequency^i^**	3	−0.233 (0.251) [.37]	−0.200 (0.433) [.65]	0.880 (0.553) [.37]	0.360 (0.230) [.14]	16	2304
Light	3	−0.059 (0.102) [.57]	0.077 (0.174) [.67]	0.232 (0.266) [.40]	0.189 (0.927) [.06]	11	1302
	4	—	—	0.208 (0.243) [.40]	0.188 (0.085) [.04]	21	1302
Medium	3	−0.055 (0.109) [.62]	−0.123 (0.188) [.52]	0.111 (0.289) [.71]	0.622 (0.100) [.54]	NS	1428
Heavy	3	−0.023 (0.200) [.76]	−0.040 (0.139) [.78]	0.221 (0.200) [.29]	0.049 (0.070) [.50]	NS	1483
**Physical activity duration^j^**	3	−0.407 (0.277) [.16]	−0.033 (0.493) [.95]	0.578 (0.761) [.46]	0.046 (0.262) [.86]	NS	2281
Light	3	−0.133 (0.102) [.29]	−0.027 (0.181) [.88]	0.058 (0.278) [.84]	0.105 (0.096) [.29]	4.0	1421
Medium	3	−0.159 (0.098) [.12]	0.153 (0.171) [.39]	0.338 (0.262) [.22]	0.029 (0.090) [.76]	NS	1373
Heavy	3	−0.037 (0.096) [.57]	−0.004 (0.016) [.98]	0.116 (0.240) [.64]	0.048 (0.082) [.57]	NS	1454
**Psychosocial Factors**							
**Self-efficacy of walking for exercise^k^**	3	−0.074 (0.056) [.19]	−0.021 (0.083) [.69]	0.267 (0.136) [.03]	−0.027 (0.045) [.36]	69	1219
	4	−0.066 (0.050) [.21]	—	0.290 (0.127) [.04]	—	97	1218
**Self-efficacy of walking or biking instead of taking a car, bus, or subway to school^k^**	3	−0.193 (0.095) [.06]	−0.106 (0.155) [.51]	0.346 (0.249) [.18]	−0.038 (0.088) [.68]	21	1455
	4	−0.204 (0.086) [.03]	—	0.373 (0.230) [.12]	—	40	1455
**Behavioral intention for physical activity^l^**	3	−0.105 (0.073) [.17]	−0.1907 (0.121) [.14]	0.364 (0.185) [.07]	0.028 (0.062) [.66]	55	1202
	4	−0.152 (0.069) [.04]	—	0.303 (0.002) [.12]	—	49	1205
**Habit strength for physical activity^l^**	3	−0.134 (0.074) [.09]	0.132 (0.116) [.91]	0.289 (0.168) [.11]	0.128 (0.044) [.04]	91	1465
	4		—	—	0.126 (0.056) [.05]	51	1460

Abbreviations: —, variable was not included in the model; NS, nonsignificant.

a Model 3 is the full model, which uses all 4 built environment characteristics of interest and controls for age and body mass index *z* score. Model 4 is the reduced model, which was constructed by using stepwise exclusion of nonsignificant effects from model 3.

b Total crime index was personal crime, murder, rape, robbery, assault, property crime, burglary, larceny, and motor vehicle theft, where 100 represents the national average.

c Counts of all unique bus and subway stops divided by the total enrollment zone area in square kilometers.

d Sum of *z* scores of land use mix, intersection density, residential population density, and retail floor area density, ranging from −14.19 to 13.20. A higher score on the index represents a neighborhood more likely to support walking.

e Proportion of school neighborhood (enrollment zone) land area covered by parks and playgrounds, ranging from 0 to 1.

f Percentage of variation in mean physical activity behaviors and psychosocial factors explained by neighborhood-level built environment characteristics, calculated as (between-school variation from model 1 minus between-school variation from model 3 OR model 4) divided by between-school variation from model 1. Model 1 examined the between-school variation in individual students’ physical activity behaviors and psychosocial factors without any explanatory variables.

g Deviance is defined as minus twice the natural logarithm of the likelihood and is used an indicator of model fit.

h β coefficient (standard error) estimated for a difference equivalent to a 1 interquartile range (75th percentile minus 25th percentile) change.

i Assessed with the questions: light intensity, “In the past week, I did things that got me up and moving”; medium intensity, “In the past week, I did things that made my heart beat a little faster”; and heavy intensity, “In the past week, I did things that got my heart beating really fast.”

j Assessed with the questions: light duration, “How long each time did I do things that got me up and moving?”; medium duration, “How long each time did I do things that made my heart beat a little faster?”; and heavy duration, “How long each time did I do things that got my heart beating really fast?”

k Assessed with the question “I am sure I can walk for exercise” and “I am sure I can walk or bike to school instead of taking a car, bus or subway.”

l Assessed with the question “I would like to do more physical activity” and “When I think about myself, physical activity is part of my daily routine.”

For boys, model 4 explained about 47% of the between-school variation in medium PA frequency, with public transportation density (β = −0.428; *P* = .02) and total crime (β = 0.193; *P* = .04) as significant predictors. Total crime was inversely associated with boys’ light PA duration (β = −0.189; *P* = .02). Total crime (β = −0.178; *P* = .03) and walkability (β = 0.179; *P* = .40) together were associated with boys’ behavioral intention for PA, explaining 31% of the between-school variation. Habit strength for PA was also associated with public transportation density (β = 0.375; *P* = 0.02) and total crime (β = −0.216; *P* = .01) in model 4 with 67% of between-school variation explained.

For girls, model 4 yielded a positive association between park access and light PA frequency (β = 0.188; *P* = .04), explaining 21% of the between-school variation. Self-efficacy for walking for exercise was negatively associated with total crime (β = −0.066; *P* = .21) and walkability (β = 0.290; *P* = .04) together, explaining 97% of the between-school variation. Total crime was inversely associated with self-efficacy for walking or biking to school (β = −0.204; *P* = .03). A positive association was observed between habit strength for PA and park access in Model 3 (β = 0.128; *P* = .04).

## Discussion

In our study of the relationship between characteristics of the built environment of school neighborhoods and students’ PA behaviors and psychosocial factors, we found that total crime and public transportation density were inversely associated with PA and psychosocial factors and that park access was positively associated with PA and psychosocial factors. Walkability alone was not associated with PA or psychosocial factors. We also observed differences by sex in relationships with built environment characteristics; for boys and girls, PA was related to the total crime, and among boys, PA was inversely associated with public transportation density.

The associations we observed are consistent with those reported in other studies of the built environment. Neighborhood crime rate and park access, in particular, appear to be robust determinants of children’s PA (6,7,17–21). Specific features of parks, such as the availability of recreational facilities and amenities, may also influence children’s PA (22). Measuring these features can identify the mechanisms through which individuals interact with their neighborhood environment.

Walkability has been inconsistently related to children’s PA. Although aspects of the walkability index, including residential population density (21) and land use mix (23), were associated with children’s PA in similar studies, we observed no significant associations in our sample. Walkability may be less influential for children because they do not frequently engage in utilitarian trips (eg, shopping) and, particularly in NYC, there are likely large numbers of potential destinations close to school. Furthermore, our sample of school neighborhoods had a higher walkability index than the citywide average, suggesting that children living in neighborhoods with lower walkability were underrepresented in this study.

We also observed public transportation density to be negatively associated with PA among boys, suggesting that the availability of subway and bus stops may influence the decision to walk to destinations for PA. Previous work in NYC has shown that educating students to purposely walk instead of using public transportation increased walking (24,25) and, elsewhere, built environment characteristics of the neighborhood appeared to moderate the effect of an intervention to promote walking for transportation among adults (26). Thus, this finding may have implications for the design and evaluation of future obesity-prevention interventions.

Although many studies have included spatial analysis in recent years, few have used a school neighborhood definition (8,9,28). Given that there is no widely accepted definition of a neighborhood, researchers have argued that the most practical definition is one that is policy relevant (27). In this study, we used the school enrollment zone as the neighborhood and assumed that this definition can serve as a proxy of the students’ activity space, or the totality of locations within which a person is exposed to and uses in a day. In NYC, it is likely that students and their parents live within the enrollment zone where they attend school, and in the small chance that they do not, they would be required to travel within it to attend school.

The within-school environment, including school size, playground availability, and frequency of physical education, may explain variation in PA (8,9). We showed that the neighborhood school environment is also a relevant predictor of PA. Researchers should consider these characteristics when evaluating school-based obesity prevention programs, because the characteristics may contribute to considerable between-school variation in PA outcomes. At worst, not accounting for these potentially confounding variables can lead to a failure to detect an intervention effect (10).

The finding that several environmental characteristics are associated both with PA and related psychosocial factors supports a dual-process framework of how environment can influence behavior. The Environmental Research Framework for Weight Gain Prevention proposes that the environment influences behavior through direct and indirect processes (29). Direct processes are spontaneous responses to environmental stimuli, for example, the automatic decision to take the bus instead of walk upon seeing an approaching bus. Indirect processes, in contrast, are mediated by cognitions about the environment, such as a decreased behavioral intention to engage in PA as a result of a high crime rate. The psychosocial factors in this study (habit strength, behavioral intention, and self-efficacy) have empirical support as mediating variables in the context of previous intervention studies (11,25). However, we did not directly assess the relationship between psychosocial factors and students’ PA behavior in our study.

Our findings are limited by a reliance on self-reported PA and a lack of demographic data at the individual level. Although we used a previously validated survey instrument, the PA frequency scale in particular had a less than optimal internal consistency reliability. A further limitation is the small sample of schools, which may be representative only of those in similar urban neighborhoods with a large proportion of black and Hispanic students eligible for free or reduced-price lunch. Although we provide the built environment characteristics of all NYC school neighborhoods for comparison, further study is warranted to determine whether these results are generalizable.

Our study provides support for policies aimed at changing the built environment surrounding urban elementary schools to encourage PA. These findings may also help design and evaluate school-based obesity prevention programs and inform policy changes that address environmental characteristics such as crime and public transportation. In instances where policy and environmental changes are infeasible, school administrators could allocate additional time for PA during the school day.
